# PP2A subunit PPP2R2C is downregulated in the brains of Alzheimer’s transgenic mice

**DOI:** 10.18632/aging.103048

**Published:** 2020-04-14

**Authors:** Waiian Leong, Wei Xu, Bo Wang, Shuaiyun Gao, Xiuyun Zhai, Cuicui Wang, Eric Gilson, Jing Ye, Yiming Lu

**Affiliations:** 1 Shanghai Ruijin Hospital, Shanghai Ruijin Hospital North, Affiliated to Shanghai Jiaotong University School of Medicine, International Laboratory in Hematology, Aging and Cancer, State Key Laboratory of Medical Genomics, Pôle Sino-Français de Recherche en Sciences du Vivant et Génomique, Shanghai, P.R. China; 2 Shanghai Ruijin Hospital North, Shanghai Jiaotong University School of Medicine, Shanghai, P.R. China; 3 Université Côte d’Azur, CNRS, INSERM, IRCAN, Faculty of Medicine, Nice, France; 4 Department of Genetics, CHU, Nice, France

**Keywords:** Alzheimer’s disease, PP2A phosphatase, tau phosphorylation, PPP2R2C

## Abstract

Targeting of PP2A suggests a close link to tau-related cognitive and functional declines. However, little is known about how the expression of PP2A subunits and PP2A activity are dysregulated in the course of AD, precluding any specific targeting strategy for restoring PP2A in AD patients. Although the PP2A heterotrimer containing the regulatory subunit PR55/Bα (encoded by the *PPP2R2A* gene) is the major tau phosphatase, the involvement of other brain-specific PP2A regulatory subunits in tau dephosphorylation remains unknown. PR55/Bγ (encoded by the *PPP2R2C* gene) is a pivotal phosphatase in the brain, and single-nucleotide polymorphisms (SNPs) of *PPP2R2C* are involved in several mental disorders. By measuring the differential spatiotemporal expression patterns of PPP2R2C in Wt and transgenic AD mice, we revealed that PPP2R2C expression is downregulated in the aged AD mouse brain as compared to the Wt mouse brain. In cultured cells, *PPP2R2C* expression regulates PP2A activity and tau dephosphorylation. These results suggest that dysregulation of PPP2R2C expression may be involved in the onset of AD and that specifically targeting PPP2R2C expression or activity is a promising strategy against brain dementia disorders, including AD and other tauopathies.

## INTRODUCTION

Dementia is a growing global health and social burden due to large aging populations. Alzheimer’s disease (AD) is responsible for 50–60% of dementia cases [1]. AD is a chronic neurodegenerative disorder characterized by progressive memory loss and inability to form new memories. Currently, more than 25 million individuals are living with AD worldwide, and this number doubles every 20 years. There are two histopathological hallmarks of AD: accumulation of amyloid plaques comprised of multiple forms of Aβ deposits, and neurofibrillary tangles of aggregated phosphorylated tau proteins [2, 3]. After numerous failures of Aβ-targeting drugs for AD, interest is growing in the therapeutic potential of targeting tau [4].

The microtubule-associated protein tau is a scaffolding protein found mainly in neuronal axons, as well as in neuronal cell bodies, dendrites, and non-neuronal cells such as astrocytes and oligodendrocytes. The major biological function of tau is that of promoting microtubule assembly and maintaining the stability of existing microtubules, which are essential for axonal transport in neurons. Under pathological conditions, tau aggregates into neurofibrillary tangles (NFTs), impairing brain function and leading to progressive neurodegeneration, a defining feature of the group of neurological disorders known as tauopathies, which includes familial frontotemporal lobar degeneration (FTLD), progressive supranuclear palsy (PSP) and corticobasal degeneration (CBD), as well as other disorders that involve tau pathology [5].

Hyperphosphorylation of tau has been shown to play crucial roles in AD, including sequestration of normal tau, disassembly of microtubules and assembly of paired helical filaments (PHFs). PP2A accounts for approximately 70% of tau phosphatase activity in the human brain and PP2A activity is significantly reduced in AD brains [6]. PP2A is the most effective phosphatase for dephosphorylating hyperphosphorylated tau isolated from AD brains. Decreasing tau phosphorylation allows recovery of its physiological function [4]. Thus, targeting of PP2A suggests a close link to tau-related cognitive and functional declines. However, little is known about how the expression of PP2A subunits and PP2A activity are dysregulated in the course of AD, precluding any specific targeting strategy for restoring PP2A in AD patients.

PP2A is a widely conserved protein serine/threonine phosphatase (PSP) that has diverse functions, accounting for the dephosphorylation of 55-70% of all serine/threonine phosphosites. It acts as a heterotrimeric protein complex consisting of a catalytic subunit (PP2Ac or C), a scaffold subunit (PR65 or A), and one of the alternative regulatory B subunits. In mammals, α and β isoforms exist for both the catalytic © and scaffolding (PR65/A) subunits; in addition, there are four B subunit families, each with several isoforms or splice variants. Thus, the functional complexity and variability of PP2A holoenzyme composition arise largely via the B subunits, which determine both the subcellular localization and a vast array of substrate specificity. PP2A is a tumor suppressor protein, and acts in a wide variety of signaling pathways, including inhibiting the activation of PI-3kinase/Akt pathway or dephosphorylation of c-Myc at Ser62, as well as NF-κB pathway, MAPK pathway, apoptosis pathway, and cell cycle progression [7]. Besides its well-known function in the brain, PP2A is also a key cardiac phosphatase that regulates diverse myocyte functions [8].

Although the PP2A heterotrimer containing the regulatory subunit PR55/Bα (encoded by the *PPP2R2A* gene) is considered to be the major tau phosphatase [6], the involvement of other brain-specific PP2A regulatory subunits in tau dephosphorylation remains unknown. Interestingly, PR55/Bγ (encoded by the *PPP2R2C* gene) is a pivotal phosphatase in the brain [9], and single-nucleotide polymorphisms (SNPs) of *PPP2R2C* are involved in several mental disorders, including ADHD, bipolar disorder and schizophrenia. Therefore, we hypothesized that PPP2R2C plays a role in tau dephosphorylation in AD. By measuring the differential spatiotemporal expression patterns of PPP2R2C in Wt and transgenic AD mice, we revealed that PPP2R2C expression is downregulated in the aged AD mouse brain as compared to the Wt mouse brain. In cultured cells, *PPP2R2C* expression regulates PP2A activity and tau dephosphorylation. These results suggest that dysregulation of PPP2R2C expression may be involved in the onset of AD and that specifically targeting PPP2R2C expression or activity is a promising strategy against brain dementia disorders, including AD and other tauopathies [10–13].

## RESULTS

### PPP2R2C regulates PP2A phosphatase activity

**Figure 1:**
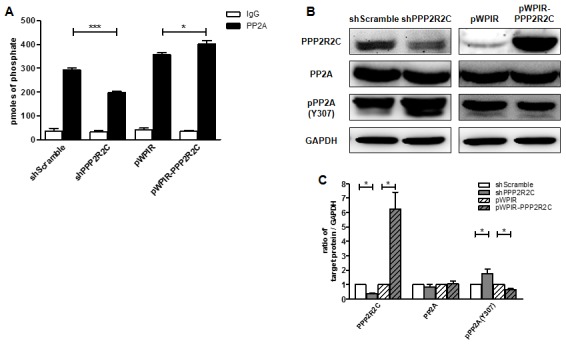
**PPP2R2C regulates PP2A phosphatase activity.** (**A**) Phosphatase activity combined with immunoprecipitation of PP2A was measured in SHSY5Y human neuroblastoma cell lines. Cells were either knocked down PPP2R2C expression by shRNA or overexpressed wildtype PPP2R2C by pWPIR-GFP lentivirus vector. Data are shown in mean+/-SEM, n=3, *p<0.05, ***p<0.001. (**B**) Representative image of immunoblots of indicated antibodies are shown in SHSY5Y cell lines after knockdown and overexpression PPP2R2C. (**C**) Quantification of the intensities of the protein bands from three independent immunoblots of (**B**). Data are shown in mean+/-SEM, n=3, *p<0.05.

First, we investigated whether PPP2R2C is essential for PP2A activity. We measured phosphatase activity combined with immunoprecipitation of PP2A in SHSY5Y human neuroblastoma cell lines. After knocking down PPP2R2C expression, we observed decreased PP2A phosphatase activity, while overexpression of wildtype PPP2R2C increased PP2A phosphatase activity (Figure 1A).

We further addressed the mechanism through which PPP2R2C regulates PP2A activity. The activity of PP2A is regulated by several post-translational modifications, including phosphorylation of Tyr or Thr, which inactivates PP2A, and methylation of the carboxyl-terminal leucine, Leu309, which activates PP2A. Interestingly, downregulation of PPP2R2C led to increased phosphorylation of the PP2A catalytic (PP2AC) domain at Tyr 307, while overexpression of PPP2R2C had the opposite effect (Figure 1B and 1C). This finding indicates that PPP2R2C regulates PP2A activity through phosphorylation of the PP2AC subunit.

### PPP2R2C expression decreases in the brains of AD mice

**Figure 2:**
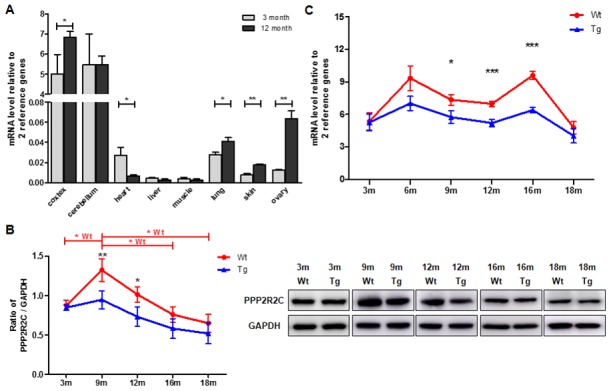
**PPP2R2C are differentially expressed in mouse brain tissues throughout lifespan.** (**A**) Quantitative RT-qPCRs for PPP2R2C were analyzed in wildtype mouse cortex, cerebellum, heart, liver, muscle, lung, skin and ovary at 2 time-points respectively (3 month and 12 month, n=9 each). Significance was tested between cortex, cerebellum, heart, lung, skin and ovary. Each measure represents the average fold-change expression of nine independent repetitions (Biological triplicate in technical RT duplicate) normalized to two housekeeping genes (β-actin and 36B4); ΔΔCt method). Mean+/-SEM with associated statistical significance are reported (*p<0.05, **p<0.01). (**B**) Representative image and the quantification from immunoblots of PPP2R2C antibody in wildtype (Wt) and transgenic (Tg) mouse cortex at different time-points of lifespan. Data are shown in mean+/-SEM, n=9 each condition, *p<0.05, **p<0.01. (**C**) Quantitative RT-qPCRs for PPP2R2C in wildtype (Wt) and transgenic (Tg) mouse cortex at different time-points of lifespan from 3 month to 18 months. Each measure represents the average fold-change expression of nine independent repetitions (Biological triplicate in technical RT duplicate) normalized to two housekeeping genes (β-actin and 36B4); ΔΔCt method). Mean+/-SEM with associated statistical significance are reported between 9, 12 and 16 month (*p<0.05, ***p<0.001).

**Figure 3:**
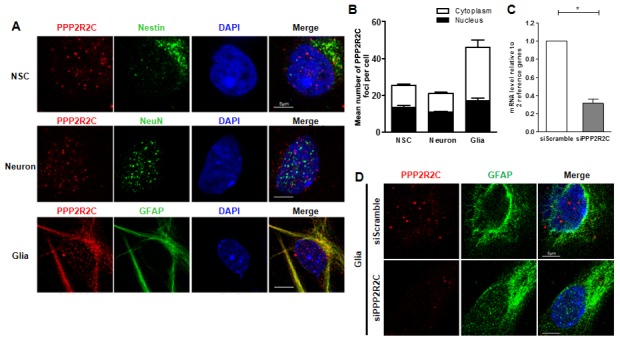
**Detection of PPP2R2C expression in different neural cells isolated from mice brains.** (**A**) Representative images of confocal sections from cultured primary cells (neural stem cell, NSC; astrocyte, Glia and neuron) isolated from wildtype newborn mouse brain, staining with of PPP2R2C antibody (red) and neural markers (green) and counterstained with DAPI (blue). (**B**) Quantification of the mean number of PPP2R2C foci per nucleus and Cytoplasm of (**A**) normalized to the number of nuclei. Data are presented at mean+/-SEM. (**C**) Quantitative RT-qPCRs analyzing PPP2R2C RNA level (n=3) and (**D**) Representative images of confocal sections staining with of PPP2R2C antibody (red) and neural markers (green) and couterstained with DAPI (blue) in glia cells isolated from wildtype newborn mouse brain after silencing of PPP2R2C. Scale bar 5 um, * p<0.05.

Next, we measured the expression pattern of PPP2R2C mRNA in the mouse brain (cortex and cerebellum), heart, liver, intestine, muscle, lung, skin and ovary at different ages. As expected, in young (3 months) and old (12 months) mice, the relative expression of PPP2R2C mRNA was much higher in the two brain tissues than in the other organs tested (Figure 2A, Supplementary Figure 1). Then, we asked whether PPP2R2C is expressed in specific cell types of the brain. We detected PPP2R2C both in the nucleus and in the cytoplasm of neural stem cells, neurons and astrocytes isolated from the brain of newborn Wt mice (Figure 3). Together with the high level of PPP2R2C mRNA in both the cortex and cerebellum (Figure 2A), these results suggest that PPP2R2C is restricted neither to a particular cell population nor to a particular region of the brain.

Then, we investigated whether the expression level of PPP2R2C mRNA and protein in the cortex varies over the lifetimes of Wt and AD (B6C3-Tg (APP^Swe^/PS1^dE9^)) mice [14]. In both genotypes, the protein level of PPP2R2C is maximum at 9 months while the increase is much less marked in AD mice and remains lower than Wt over life time (Figure 2B). Interestingly, the PPP2R2C expression level does not differ between Wt and AD mice in young animals (3 months, Figure 2B) and becomes differentially regulated between 3 and 9 months, i.e. at the time where the AD symptoms appear [15]. The mRNA level of PPP2R2C is also reduced in AD mice as compared to age-matched Wt mice (Figure 2C). Notably, as compared to the protein expression level, there is a second peak of PPP2R2C mRNA expression later in life (16 months, Figure 2C), suggesting a differential post-transcriptional regulation of PPP2R2C in aging mice.

In summary, PPP2R2C expression is significantly downregulated after brain maturation in AD mice.

### PPP2R2C is required for tau dephosphorylation

**Figure 4:**
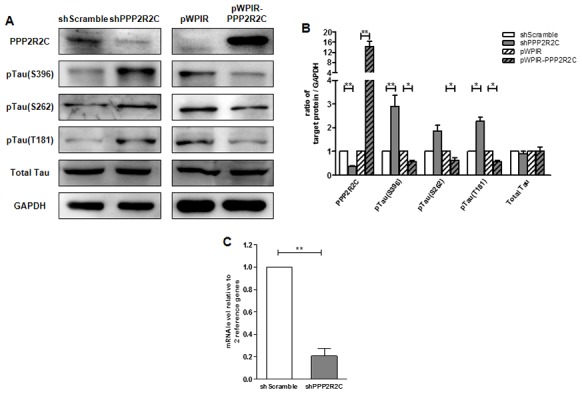
**PPP2R2C level regulates Tau phosphatase levels. (A)** Representative image of immunoblots of indicated antibodies are shown in SHSY5Y cell lines after knockdown and overexpression PPP2R2C. (**B**) Quantification of the intensities of the protein bands from three independent immunoblots of (**A**). (**C**) Quantitative RT-qPCRs analyzing PPP2R2C mRNA level in SHSY5Y cell lines after knockdown PPP2R2C. Data are shown in mean+/-SEM, n=3, *p<0.05, **p<0.01.

Finally, we asked whether *PPP2R2C* could contribute to tau dephosphorylation. Knockdown of PPP2R2C led to increased levels of phospho-tau at S396, S262 and T181 (Figure 4). Overexpression of PPP2R2C inhibited tau phosphorylation. Thus, PPP2R2C is required for tau dephosphorylation. This regulation of tau dephosphorylation by PPP2R2C is not dependent on the particular cell line used since it is also observed in 293T cells, a human renal epithelial cell line (Supplementary Figure 2A–2C).

## DISCUSSION

Given the lack of efficacy of current amyloid-β-targeted therapies for Alzheimer’s diseases (AD), interest is growing in tau as a potential alternative target. PP2A is the main phosphatase involved in dephosphorylating tau and therefore constitutes a valuable target in the treatment of AD and other tauopathies. However, PP2A plays pleiotropic roles in various organs, indicating the need for therapeutic strategies specific to PP2A enzyme in the brain, to avoid severe secondary effects. Our results reveal that PPP2R2C is a brain isoform of the regulatory subunit of PP2A exhibiting a reduced expression in the brains of adult AD mice. Interestingly, the differential expression of PPP2R2C between Wt and AD mice starts during adulthood when the first AD symptoms appear in these mice. We also show, using different human cell lines, that PPP2R2C is involved in tau dephosphorylation. Together, these observations reveal PPP2R2C as an interesting candidate to contribute to AD in human.

A limitation of our study is the use of only one mouse model of Alzheimer’s disease, even if it is widely validated in the field [16, 17]. Measuring the expression and activity of PPP2R2C in other mouse models will open new opportunities to deepen our understanding of the pathway of Tau phosphorylation regulation unveiled here.

We also noted a low but detectable level of PPP2R2C mRNA in lung, skin and ovary, in Wt mice which increases with age. Whether these variations of mRNA level of PPP2R2C expression contribute to aging is an interesting question for further studies.

In order to consider PPP2R2C as a valuable target to prevent and/or treat AD patients, our findings have to be confirmed in post-mortem human brains of AD patients and in genetic mouse models of PPP2R2C downregulation. A possible scenario would be to increase the expression of PPP2R2C in patients presenting the first symptoms or suspected to develop an AD disease. As PPP2R2C does not carry an intrinsic enzymatic activity, its pharmacological targeting might be difficult. An alternative strategy could be to target PPP2R2C regulators. Interestingly, we previously showed that PPP2R2C is positively regulated by the telomeric protein TRF2 [18], whose expression decreases in AD patients [19]. This, together with the established role of PP2A in the dephosphorylation of key DNA damage response (DDR) factors, suggests that, in addition to tau dephosphorylation, PPP2R2C attenuates the DDR in the brain, a hallmark of aging. This raises the possibility to intervene by activating TRF2. This would have the double advantage to increase PPP2R2C expression and to protect telomeres, which are suspected to play a role in AD [19, 20].

For all these reasons, PPP2R2C represents a promising multi-hit target to prevent and treat AD patients.

## MATERIALS AND METHODS

### Cell lines, media and reagents

293T cells and SHSY5Y cells (human neuroblastoma cell line) were obtained from cell resources center of Shanghai Life Research Institute. The cell were cultured in DMEM medium (Gibco, MA, USA) supplemented with 10% fetal calf serum (FBS) and 1% (v/v) penicillin-streptomycin (Sangon, China) at 37°C, 5%CO2 cell culture incubator. The cell culture media were changed every 3 days until the cells were confluent.

### Lentiviral packing and transfection

To create the lentiviruses, 8.6 μg of pWPIR-GFP vector expressing empty vector, PPP2R2C; or pLKO.1 vector expressing PPP2R2C shRNA or non-coding shRNA, 8.6 μg of pCMVΔR8.91 and 2.8 μg of pCMV-VSV-g were introduced into 5×10^6^ 293T cells in DMEM, supplemented with 10% FBS at 37° in 5% CO2 in 10 cm dishes, through calcium phosphate mediated transfection (Promega E1200, USA). Virus containing supernatants were collected after 48 hours and 72 hours, passed through a 0.45 μm Millipore filter and used to infect target cells. The efficiency of infection was determined by flow cytometry analysis of GFP expression 3 days or puromycin selection 1 week after infection.

### Lentivirus production and infection

Lentiviruses were produced by transient calcium phosphate transfection of 293T cells with the virus packaging plasmids, p8.91 and pVSVg, as well as with the lentiviral expression vector that contained the sequence of interest. Titration was performed approximately 10 days after infection by means of puromycin (1 μg/ml) selection of clones. The following shRNA plasmids were purchased from Sigma and used for lentivirus production: pLKO-shScramble, pLKO-shPPP2R2C. The construct containing full-length PPP2R2C was designed and performed in the pWPIR lentivirus vector system. Infection with various shRNAs or overexpression vectors was performed for a minimum of 4 days, and depending on experiment, cells were kept in culture for up to 10-15 days after infection. Efficiency of each shRNA was checked routinely by RT-qPCRs or western blotting.

### Mice and tissue preparation

APP^Swe^/PS1^dE9^ (The Jackson Lab, No. 004462) Tg mice and their Wt littermates used in this study at the age of 3, 6, 9, 12, 16 and 18 months were bred by male heterozygote and female Wt mice. The maintenance, breeding and staging were performed in accordance with the Laboratory Animal Care Guidelines approved by the animal committee of Shanghai Jiaotong University School of Medicine. The mice were anesthetized and perfused with ice-cold 0.1M PBS, and then the cerebral cortex, cerebellum, heart, liver, muscle, lung, skin and ovary were dissected, and tissues were used to prepare RNA and/or protein.

### Primary neural cell, media and reagents

Primary neural cell were isolated from newborn Wt mouse brains at 0-1 days old. The brain tissues were placed to cold PBS, then cerebral cortices and hippocampus were carefully dissociated. Cultures for neural stem cell were incubated in NeuroCult NSC basal medium with NSC Proliferation supplement, 20 ng/ml EGF, 20 ng/ml bFGF, 2ug/ml heparin and 1% Pen-Strep. Cultures for neuron were incubated in serum-free Neurobasal medium supplemented with B-27, L-glutamine and 1% Pen-Strep. Cultures for astrocytes (Glia) were incubated in DMEM and Ham’s nutrient mixture F-12 (1:1) medium supplemented with 10% FBS, 1xglutamax and 1% Pen-Strep. A single-cell suspensions was prepared by trypsinization or PDD (Papain, DNase I, Dispase) and washed with corresponding culture medium, then plated in 24 well plate at a density at 1*10^5 cells/ well. All the primary cells were maintained at 37°C in a 5% CO2 incubator and split every 3–5 days and then the cells were ready for subsequent study.

### Immunofluorescence assay

Cells were grown onto glass cover slips and fixed for 10 min with 3% formaldehyde in PBS. Cells were then permeabilized with 0.5% Triton X-100 for 10 min and incubated for at least 2h at room temperature in blocking solution (0.5% Triton X-100 and 4%BSA in PBS). Cells were then stained overnight at 4°C in blocking solution containing the respective primary antibodies (PPP2R2C, 1:100, SantaCruz, sc100417; Nestin, 1:100, Abgent, AP2020; NeuN, 1:200, Millipore, ABN78; GFAP, 1:200, Abcam, ab53554). After 0.1% Triton X-100 in PBS washes, cells were incubated for 1 hour at 37°C with the corresponding secondary antibodies (Alexa Fluor, 1:400, Invitrogen) in blocking solution. Finally, cells were mounted with DAPI (Vectashield, Vector Laboratories). Images were obtained with a Leica SP8 confocal microscope.

### Real-time RT-qPCRs

Total RNA was isolated from organs and cells using the Trizol reagent (Invitrogen, USA). The RNA pellet was dissolved in diethyl pyrocarbonate-treated H2O. First-strand cDNA synthesis was performed with 0.5 μg of total RNA using oligo(dT) primers and Superscript I reverse transcriptase (Takara, Japan) for mouse tissues. 1 μl of the reaction product was taken for real time RT-qPCRs amplification (ABI Prism 7000, Applied Biosystems) using a commercial SYBR® Green kit (Tiangen, China). Primer sequences were available on request. Expression of each gene was normalized to the respective beta-actin, and Rplp0 (36B4) expression.

### Western blots

Cells and Mouse cortex were lysed with the ice cold lysis buffer containing 1xRIPA (Beyotime Biotechnology, China), 1mM Phenylmethylsulfonyl fluoride (PMSF, Beyotime Biotechnology), a protease inhibitor mixture (Roche Molecular Biochemical, Switzerland), and a phosphatase inhibitor cocktail (Roche Molecular Biochemical, Switzerland) for 5 min. The lysate was centrifuged at 12,000× g for 30 min at 4°C, and the supernatant fraction was used for western blot analysis. 30-50ug of protein samples were subjected to 10%SDS–polyacrylamide gel electrophoresis. The gels were transferred onto PVDF membranes (Millipore, USA). The membranes were incubated for 1h at room temperature in blocking solution (5% milk in TBS buffer containing 0.1% (v/v) Tween-20 (TBST)), and then were incubated overnight at 4°C in blocking solution containing the following antibodies: mouse monoclonal anti-PPP2R2C (1:500, Santa Cruz, sc100417), mouse monoclonal anti-PPP2R2C (1:1000, Abcam, ab58153), mouse monoclonal anti-GAPDH (1:2500, Aogma, 9600), rabbit polyclonal anti-Tau(1:2500, Abcam, ab64193), rabbit monoclonal anti-Tau phospho S396(1:10000, Abcam, ab109390), rabbit monoclonal anti-Tau phospho S262(1:2500, Abcam, ab92627), rabbit polyclonal anti-Tau phospho T181(1:2500, Novus bio, NB100-82245), rabbit monoclonal anti-PP2A(1:2500, epitomics, 1512-1), rabbit monoclonal anti-PP2A phospho Y307(1:2500, epitomics, 1155-1). For enhanced chemiluminescence (ECL) detection, HRP-conjugated rabbit antibody (1:2500, beyotime, A0208) and mouse antibody (1:2500, cwbio, cw0102) were used. The intensities of the protein bands were scanned using the ImageQuant LAS 4000 System (GE healthcare, UK).

### PP2A activity assay

Cells were lysed with the ice cold lysis buffer 20mM imidazole-HCl, 2mM EDTA, 2mM EGTA, pH 7.0 with 10ug/ml each of aprotinin leupeptin, pepstatin, 1mM benzamidine, and 1mM PMSF and centrifuged at 2000 x g for 5 min at 4°C. The supernatants were used to assay phosphatase activity using a PP2A Immunoprecipitation Phosphatase Assay System kit (Millipore, 17-313) in accordance with the manufacturer’s protocols. After adding phosphopeptide and Ser/Thr assay buffer, parts of the samples were added 6nM Okadaic acid (Beyotime Biotechnology) to inhibit PP1/PP2A activity. The 96-well plates were measured the O.D. at 650nm using Power Wave XS2 (BioTek, Vermont, USA) and the absorbance values of samples compare to negative controls containing no enzyme.

### Statistical analysis

The results were expressed as mean +/- SEM; the SEM values for these groups were analyzed on the basis of three independent experiments. Parametric variables of normal distribution were analyzed by either a two-tailed Student’s t-test or ANOVA with Bonferroni test as post-hoc test. Results were considered significant at p<0.05. Statistical analysis was performed using the SAS 8.2 statistical package (SAS Institute Inc., USA) and GraphPad Prism 5.01 (GraphPad software Inc., USA).

### Primers used to determine PPP2r2c, housekeeping genes and tissue specificity expression

─ human

PPP2R2C

forward: 5’-CACTCCTGTCCACCAACGAT-3’,

reverse: 5’-TCACCTCCACCATCAGATCC-3’;

Rplp0 (36B4)

forward: 5’-AACTCTGCATTCTCGCTTCCT-3’,

reverse:5-ACTCGTTTGTACCCGTTGATG-3’;

beta-actin

forward: 5’-TCAAGATCATTGCTCCTCCTGAGC-3’,

reverse: 5’-AACGCAACTAAGTCATAGTCCGCC-3’;

─ mouse

PPP2R2C

forward: 5’-GATTACCGAACGAGACAAGAGG-3’,

reverse: 5’-GAGATGGAGTTGATGTGGTAGG-3’;

Rplp0 (36B4)

forward: 5’-AGATTCGGGATATGCTGTTGGC-3’,

reverse:5-TCGGGTCCTAGACCAGTGTTC-3’;

beta-actin

forward: 5’-AGAGCTATGAGCTGCCTGA-3’,

reverse: 5’-GGCATAGAGGTCTTTACGGATG-3’;

TUBB3 (Tubulin beta 3, brain)

forward: 5’- AGCAGTTCACAGCCATGTT-3’,

reverse: 5’- CCGATTCCTCGTCATCATCTTC-3’;

MYH6 (Myosin heavy chain 6, heart)

forward: 5’- GGATCCACTTTGGAGCTACTG-3’,

reverse: 5’- GCGTAGTCGTATGGGTTGTT-3’;

ALB (Albumin, liver)

forward: 5’- TTAGTGAGGTGGAGCATGAC-3’,

reverse: 5’- GTCTCAGCAACAGGGATACA-3’;

SGCD (Sarcoglycan, delta (dystrophin-associated glycoprotein), muscle)

forward: 5’- CGACCAGGTAATGCCCTATAC-3’,

reverse: 5’- ACGACCACTTCACTGTCATC-3’;

KRT5 (Keratin 5, skin)

forward: 5’- TGACACATCTGTGGTCCTTTC -3’,

reverse: 5’- CTGTTGCAGCTCCTCATACTT -3’;

SFTPA1 (surfactant associated protein A1, lung)

forward: 5’- AAGGGAGAGCCTGGAGAAA-3’,

reverse: 5’- AGTTGACTGACTGCCCATTG-3’;

OSAP (Ovary-specific acidic protein, ovary)

forward: 5’- CAGTCAGTGCTGGTGGATATT-3’,

reverse: 5’- CCGATTCAGCTTCCGTTACA-3’;

## Supplementary Material

Supplementary Figures
