# Supplementary material for: PP2A subunit PPP2R2C is downregulated in the brains of Alzheimer’s transgenic mice
Source: Aging (Albany NY). 2020 Apr 14;12(8):6880–90. doi: 10.18632/aging.103048 (PMC7202491; doi:10.18632/aging.103048)
Supplement: Supplementary Figures [file aging-12-103048-s001..pdf]

SUPPLEMENTARY FIGURES

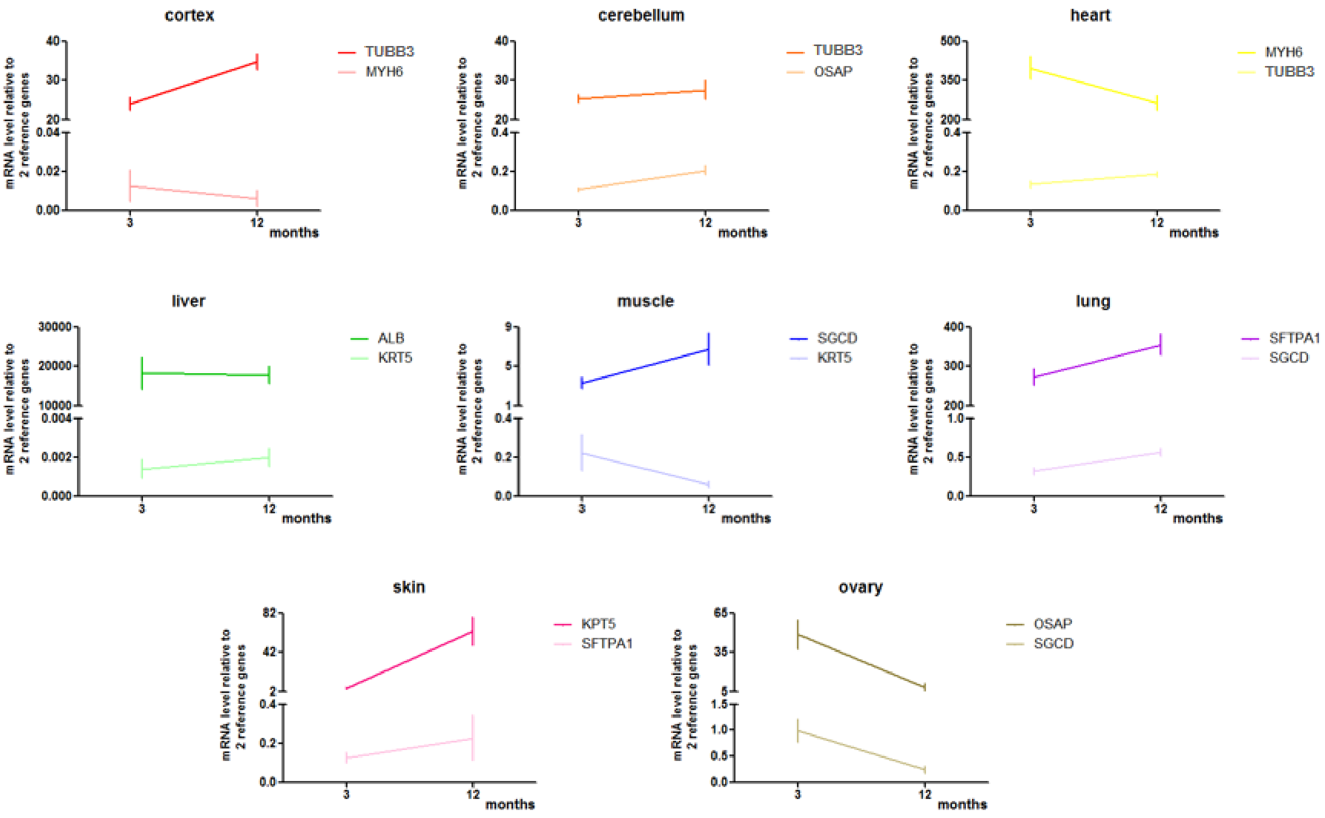

Supplementary Figure 1. Expression of tissues specific markers in the dissected organs from wildtype mice at 3 and 12 months. Quantitative RT-qPCRs for different mice organs was addressed by different tissue specific primers, including cortex, cerebellum, heart, liver, muscle, lung, skin and ovary from 3 months and 12 months, n=9 each. Data are presented at mean+/-SEM.

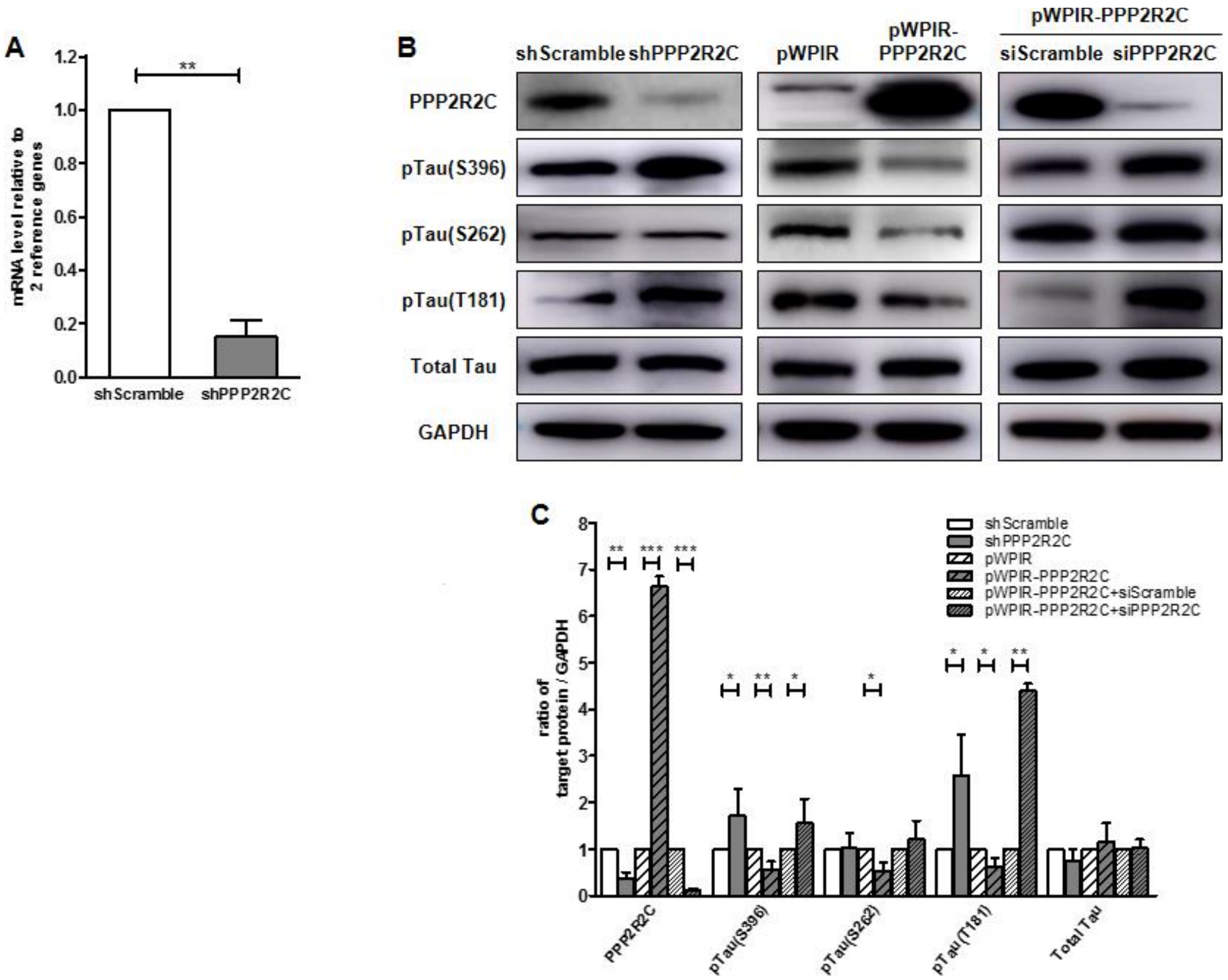

Supplementary Figure 2. Specific regulation of PPP2R2C level in 293T cells triggers variation of Tau phosphorylation. (**A**) Quantitative RT-qPCRs analyzing PPP2R2C RNA level in 293T cells after knockdown of PPP2R2C (n=3). (**B**) Representative images of Immunoblots of indicated antibodies are shown in 293T cell line after knockdown and overexpression PPP2R2C. (**C**) Associated quantifications in three independent experiments of (**B**). All data are presented at mean+/-SEM , n=3, * p<0.05, ** p<0.01, *** p<0.001.
